# Application of response surface methodology and green carbon dots as reducing agents in speciation of iron†

**DOI:** 10.1039/c7ra12139c

**Published:** 2018-01-09

**Authors:** Masoud Shariati-Rad, Tahereh Mohseninasab, Fatemeh Parno

**Affiliations:** Department of Analytical Chemistry, Faculty of Chemistry, Razi University Kermanshah Iran mshariati_rad@yahoo.com +98 833 4274559

## Abstract

Herein, for the first time, we used a green synthetic approach, *via* the hydrothermal treatment of grape and onion without any functionalization, to produce reducing carbon dots (CDs). The method has the advantages of low cost, easy operation and being environmentally friendly. The as-synthesized grape and onion CDs were characterized by UV-Vis spectrophotometry, spectrofluorimetry, FTIR spectroscopy and transmission electron microscopy (TEM). Interestingly, it was found that the synthesized CDs could reduce Fe^3+^ to Fe^2+^. Based on this finding, a method based on complexation with 1,10-phenanthroline was introduced for determination of Fe^3+^ and total iron in water samples. A response surface methodology was employed to explore the factors influencing the response, *i.e.* concentration of 1,10-phenanthroline and concentration of as-synthesized CDs. The proposed method provides a simple and sensitive colorimetric approach to detect Fe^3+^ over a wide linear range of 4.6–160 μM with a low detection limit of 0.1 μM. Moreover, for the first time, the reducing strength of CDs was estimated by the well-known Prussian blue assay.

## Abstract 2

Herein, green synthetic approach was used to produce reducing carbon dots (CDs) and for the first time, corresponding reducing strengths were estimated.

## Graphical abstract

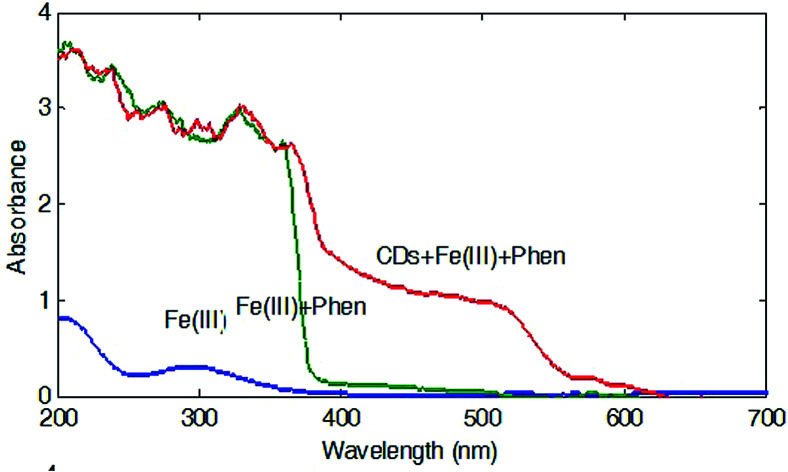

## 1. Introduction

The safety of drinking water is very important in public health. The United States and the World Health Organization have established well-defined standards for the purity of drinking water. For example, U.S. Federal Regulations limit the amount of iron to less than 0.3 ppm (0.3 mg L^−1^) in municipal drinking water. Although iron is only toxic at very high concentrations, it acts as a useful surrogate for other heavy metals, whose presence in drinking water is a real danger to public health.

The International Standards Organization (ISO) suggests a spectrophotometric method based on the formation of a colored complex between Fe^2+^ and 1,10-phenanthroline^1^ for determination of total dissolved iron in water samples. Since in the water samples, iron is present predominantly as Fe^3+^, it is necessary to first reduce Fe^3+^ to Fe^2+^.

Sequential determination of Fe^2+^ and Fe^3+^ includes firstly, complexation of Fe^2+^ with 1,10-phenanthroline in optimal conditions and secondly, reduction of Fe^3+^ to Fe^2+^ and then complexation of total iron with 1,10-phenanthroline. The difference between the results of two steps would provide the amount of Fe^3+^ and the results of the first step can be used to obtain the amount of Fe^2+^. For the reduction step, several reducing agents including sulfite,^2^ ascorbic acid,^3–5^ thioglycolic acid,^6^ photoreduction^7^ and predominately, hydroxylamine^8,9^ have been used. Evidently, these reduction processes involve using in most cases toxic chemical compounds which is against principles of green chemistry. Because of their non-toxicity, environmentally friendly nature, biocompatibility and fast response times, carbon dots (CDs) has been widely used in bio-related studies, and also in designing catalytic biosensors.^10–13^

Based on fluorescence property, CDs have been used to sensitive detection of a diverse array of salt ions specially Fe^3+^.^14–17^ It should be pointed out that in these studies, detection of metal ions is generally based on quenching CD fluorescence by metal ions including Fe^3+^. These methods usually have low selectivity. However, in a work reported by Iqbal *et al.*,^18^ a CD based sensor with 1,10-phenanthroline in its surface was prepared and used to selective and sensitive determination of Fe^3+^ and Fe^2+^. The sensor can only result in the total iron.

Heretofore, reducing ability of CDs has been mainly used in formation of Ag or Au nanoparticles and subsequent sensing of species like Ag^+^ or biothiols.^19–25^ It has been found that CDs can act as reducing agent for synthesis of AgNPs.^20–24^ The as synthesized NPs have been applied for determination of cysteine (Cys), homocysteine (Hcy) and glutathione (GSH).^20^ Au@C-dot has also been synthesized based on the reducing activity of CDs.^25^ The Au@C-dot composite can be applied as a colorimetric and fluorometric sensor for biothiols including amino acids, peptides, proteins and enzymes.^25^ Based on the reducing ability of CDs, an alloy nanocomposite with CD (AuAg@C-dots) was prepared and used for chlorine assay.^26^

Gao *et al.*^27^ observed that Ag^+^ exhibits an enhancement effect on the photoluminescence of synthesized CDs, which can be attributed to the reduction of Ag^+^ to silver nanoclusters (Ag^0^) on the surface of the CDs. Hg^2+^ detection was reported by UV-Vis absorbance changes of Ag@C-dots upon addition of Hg^2+^.^19^

It is not common to use carbon-based nanomaterials as the reducing agent in determination of other compounds. To the best of our knowledge, the metal ion detection and determination based on reducing ability of CDs has rarely been reported. As an example, the spectrofluorimetric detection of Ag^+^ based on its reduction by CDs has been reported by Gao *et al.*^27^

However, our aim in this work is to use CDs as reducing agent instead of toxic chemical compounds in sequential determination of Fe^2+^ and Fe^3+^. Moreover, we use a green method for preparing CDs.

## 2. Experimental

### 2.1. Materials

CDs were freshly prepared by appropriate amount of solid in deionized water. Iron(iii) chloride hexahydrate (FeCl_3_·6H_2_O), iron(ii) chloride hexahydrate (FeCl_2_·6H_2_O), 1,10-phenanthroline, hydrochloric acid and ethanol were all from Merck (Darmstadt, Germany). For preparation of 1,10-phenanthroline solution, hydrochloric acid (0.5 mol L^−1^) was used. Deionized water was used throughout this study as solvent.

For Prussian blue assay, gallic acid (3,4,5-trihydroxybenzoic acid monohydrate) was purchased from Sigma-Aldrich (Taufkirchen, Germany). Analytical reagent grade potassium hexacyanoferrate(iii) (K_3_[Fe(CN)_6_]) and sodium dodecyl sulfonate (SDS) were purchased from Merck (Darmstadt, Germany).

### 2.2. Apparatus

Recording of the UV-Vis spectra in the spectral range of 200–600 nm was performed by an Agilent 8453 UV-Vis spectrophotometer equipped with diode array detector, with 1 cm path-length quartz cells.

For characterization of synthesized CDs, a Zeiss EM10C transmission electron microscope (TEM), an Alpha FTIR spectrometer (Bruker, Germany) and a Jasco FP_6200 spectrofluorimeter equipped with a Jasco ECT_272T temperature controller were employed.

### 2.3. Procedure for synthesis of carbon dots

A large number of methods have been reported for preparing CDs, therein, hydrothermal and solvothermal methods are simple. Hence, in this work, hydrothermal method was employed with grape and onion as precursors. CDs were synthesized by simply heating grape and onion juices. In a typical procedure, the grape or onion (*ca.* 80 g) was cut into small pieces and turned into a paste with 100 mL of water and filtered. Then 20 mL of the filtrate was taken with 20 mL of ethanol in a 60 mL Teflon-lined pressure vessel and heated at constant temperature of 150 °C in an oven for 4 h. After cooling at room temperature, a dark brown product was obtained. The solid product was dissolved in 20 mL of water and the residue was separated by filtration. 50 mL of ethanol was added into the aqueous filtrate and centrifuged (3000 rpm) for 2 min under ambient conditions to separate the large particles. The solvent was evaporated at room temperature under vacuum to obtain CDs. The yield of synthesis was 16.2% and 12% for grape and onion, respectively.

### 2.4. Response surface methodology for exploration of the reaction between Fe^3+^ and 1,10-phenanthroline in the presence of CDs

Design of experiment is useful for providing statistical models which help in understanding the interactions between the factors that have been explored. In designing experiment, response surface methodology (RSM) has been found as a useful tool to develop quadratic regression models, it also quantifies the relationship between the controllable input factors and the obtained response surfaces.^28,29^

Factors considered for the reaction of Fe^3+^ with 1,10-phenanthroline in the presence of CDs are concentration of CDs (*x*_1_) in mg L^−1^ and concentration of 1,10-phenanthroline (*x*_2_) in mol L^−1^. Levels of the factors in the experiments designed based on RSM and corresponding responses are shown in Table S1.† Response is the absorbance of the complex between reduced Fe^3+^ (Fe^2+^) and 1,10-phenanthroline at 510 nm.

### 2.5. Procedure for calibration

To obtain calibration curve, different concentrations of Fe^3+^ were added to 1.7 mL aqueous solutions of CDs in 5 mL volumetric flasks. After 2 min, 1,10-phenanthroline (in 0.5 mol L^−1^ hydrochloric acid) was added. After completing the flasks to the mark, concentration of CDs and 1,10-phenanthroline are 1700.0 mg L^−1^ and 9.00 × 10^−3^ mol L^−1^, respectively.

### 2.6. Procedure for analysis of real water samples

Samples were analyzed within the same conditions and procedure as calibration samples. Wastewater was collected from different sites of the Qar-e-Sou River, Kermanshah, Iran. Mirage water was collected from different sites of the Niloufar mirage, Kermanshah, Iran. Tap water was collected without adding any preservative. Then, water samples were filtered through a Whatman no. 41 filter paper. For the analysis of the real samples, a volume equivalent to 1.7 mL of the stock solution of CDs was transferred to 5 mL volumetric flask containing 2.3 mL of the prepared water sample. After 2 min, 1.0 mL of 1,10-phenanthroline was added to the above mixture. Concentration of CDs and 1,10-phenanthroline are those for calibration samples. The spectra of the samples were then recorded against proper blank. For each sample, six replicates were measured and mean of the predicted concentrations was reported.

### 2.7. Prussian blue assay

3 mL of 0.1 mol L^−1^ of FeC1_3_ in 0.1 mol L^−1^ hydrochloric acid was added to the 1000 mg L^−1^ solution of CDs, followed by addition of 3 mL of 0.008 mol L^−1^ of K_3_Fe(CN)_6_. Then, after 10 min, the absorbance of the mixture was recorded at 720 nm against proper blank. It must be mentioned that calibration curve was obtained by the above procedure except using different concentrations of gallic acid instead of CDs. It must be mentioned that for stabilization of PB, to the all solutions, SDS (0.03 mol L^−1^) was added.

## 3. Results and discussion

### 3.1. Characterization of the synthesized CDs

At first, CDs were synthesized by carbonization of grape and onion which contains carbohydrates, sugars and polyphenolic compounds as the carbon precursors.

**Fig. 1:**
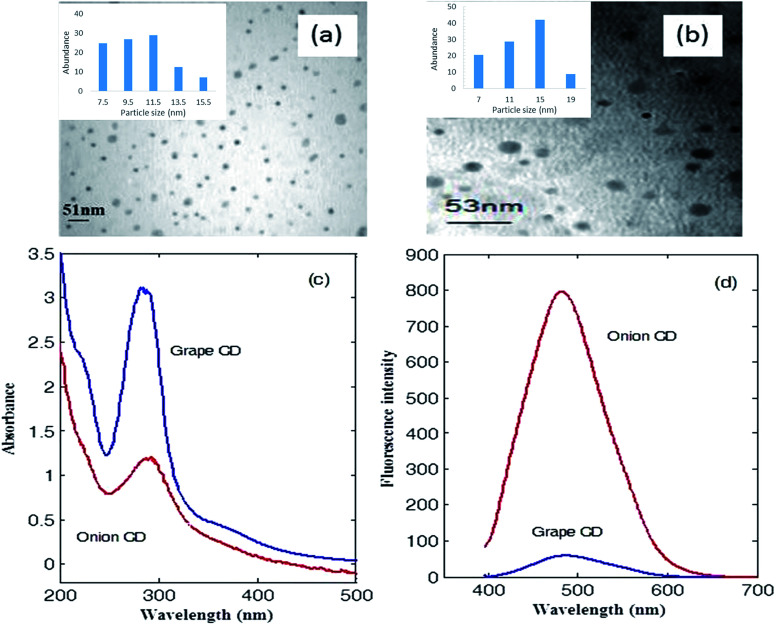
TEM images of as-synthesized (a) grape CD and (b) onion CD. UV-Vis (c) and fluorescence (d) spectra of the as-synthesized CDs. Fluorescence spectra have been obtained by excitation at 390 nm. Insets in panels (a) and (b) show the distribution of the sizes of the synthesized CDs.

Images captured by transmission electron microscopy (Fig. 1a and b) shows that the synthesized CDs are mostly spherical dots. These dots are well separated from each other with average sizes of 12.1 and 13.3 nm for grape and onion CDs, respectively.

UV-Vis absorption spectrum and fluorescence spectrum of CDs in water can be seen in Fig. 1c. Grape CDs show a main absorption band with maximum located at 284 nm which develops to about 500 nm. Shoulders at about 222 and 370 nm can also be observed in the UV-Vis spectrum of grape CD. For onion CD, the maximum absorption appears at 290 nm and shoulders appear at 220 and 360 nm (see Fig. 1c). The main absorption band of the two synthesized CDs can be attributed to the n–π* transition of C=O.^30^ The low intensity band at about 220 nm which appears as a shoulder represents the π–π* transition of C=C in aromatic structure.

An intense fluorescence spectrum was observed for the onion CDs after excitation at 390 nm with maximum intensity at 482 nm (see Fig. 1d). Grape CD also fluoresces after excitation at 390 nm (with maximum intensity at 486 nm). However, its intensity is much lower (see Fig. 1d).

For characterization of the surface groups of the synthesized CDs, FTIR spectra were recorded. Fig. S1† shows the FTIR spectrum of the as-synthesized CDs. The weak absorption band at 1703 cm^−1^ is attributed to the C=O stretching band of the carboxylic acid groups conjugated with condensed aromatic carbon, while the broad absorption band at ∼3400 cm^−1^ is assigned to –OH groups.^31^ The absorption band for C=O stretching in the region 1870 to 1600 cm^−1^ is perhaps the easiest band to recognize in IR spectrum and is extremely useful in analysis of carbonyl compounds.

The band at 1635 cm^−1^ for grape CD (1637 cm^−1^ for onion CD) is assigned to C=C stretching vibration. These results indicate that the synthesized CDs have an aromatic skeleton.^32,33^ The bands at 2118 cm^−1^ and 2128 cm^−1^ can be attributed to C–N vibration for grape and onion CDs, respectively.^34^ In-plane vibration of C=C for grape and onion CDs can be seen at 1450 cm^−1^ and 1416 cm^−1^, respectively.

The C–O stretching in phenols/alcohols occurs at a lower frequency range 1250–1000 cm^−1^. The coupling of C–O absorption with adjacent C–C stretching mode, makes it possible to differentiate between primary (∼1050 cm^−1^), secondary (∼1100 cm^−1^) and tertiary (∼1150 cm^−1^) alcohols and phenols (∼1220 cm^−1^). For the as-synthesized CDs, this characteristics band can be observed at 1075 and 1052 cm^−1^, for grape and onion CDs, respectively. The results are in accordance with the related observations in UV-Vis spectra. The observed hydrophilic –OH groups enable the as-obtained CDs to be well-dispersed in aqueous media.

### 3.2. Reducing ability of the synthesized CDs

The reducing groups on the surface of CDs, such as hydroxyl ones, endow function of CDs as reducing agents and have been used as nucleation centers for the nucleation and growth of metallic nanoparticles and act as a stabilizer to prevent aggregation of nanoparticles derived from metal salts.^20^ However, in the present work, it was found that the synthesized CDs show a novel property: the as-synthesized CDs can be used as reducing agent to reduce Fe^3+^ to Fe^2+^.

**Fig. 2:**
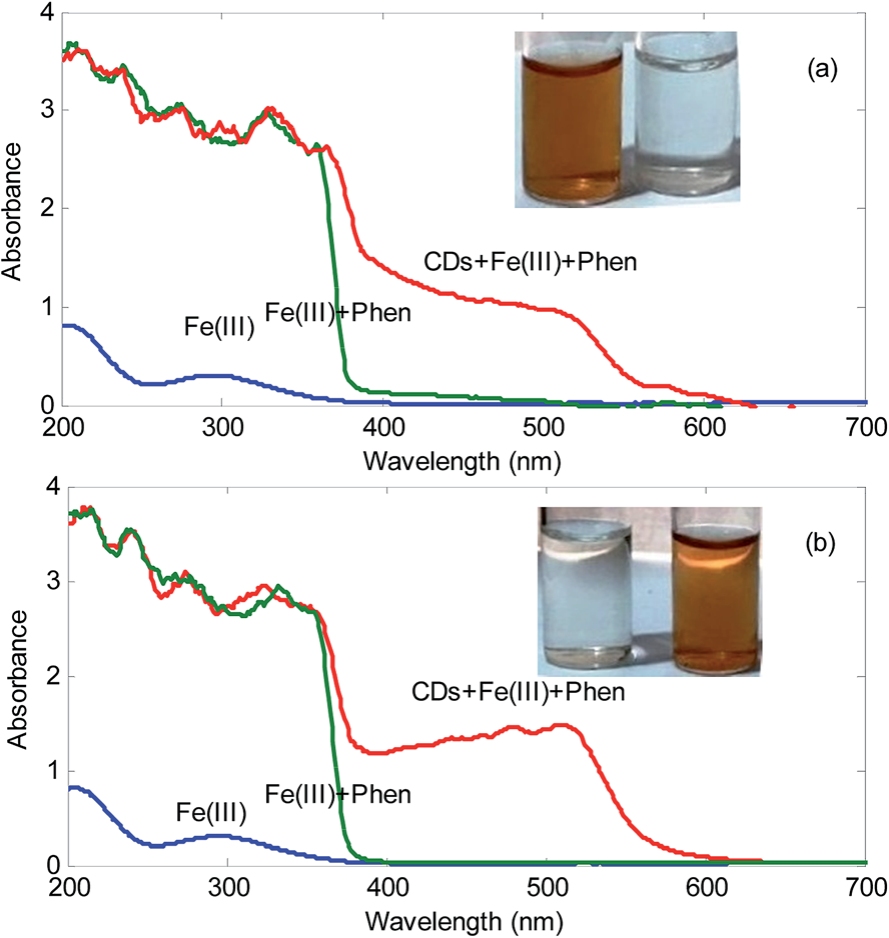
Spectra of Fe^3+^, Fe^3+^ plus 1,10-phenanthroline and Fe^3+^ plus 1,10-phenanthroline in the presence of (a) grape CDs and (b) onion CDs. Concentration of Fe^3+^, 1,10-phenanthroline and CDs are 1.20 × 10^−4^ mol L^−1^, 3.90 × 10^−5^ mol L^−1^ and 1700.0 mg L^−1^, respectively. Insets show the color changes of the mixtures in the presence of CDs.

Fig. 2 shows the spectra of the mixture Fe^3+^ plus 1,10-phenanthroline in the presence and absence of the synthesized CDs. As can be seen, in the absence of CDs, it cannot be seen the absorption peak at about 500 nm. However, in the presence of CDs, a peak located at 500 nm is observed. It must be mentioned that this peak is characteristics of the formation of complex between Fe^2+^ and 1,10-phenanthroline. Moreover, a color change from colorless to orange in the solutions is observed. This simply confirms that the synthesized CDs possess reducing ability. In published studies related to reducing ability of CDs, mainly Ag and Au@CDs composites have been prepared.^19–25^

The function of the reduction using CDs as the reducing agent can be related to the hydroxyl groups (–OH) on the surface of the synthesized CDs. Fe^3+^ can be reduced to Fe^2+^ and at the same time, the –OH converts to carbonyl groups (C=O).

### 3.3. Influence of time in reduction of Fe^3+^ by CDs

**Fig. 3:**
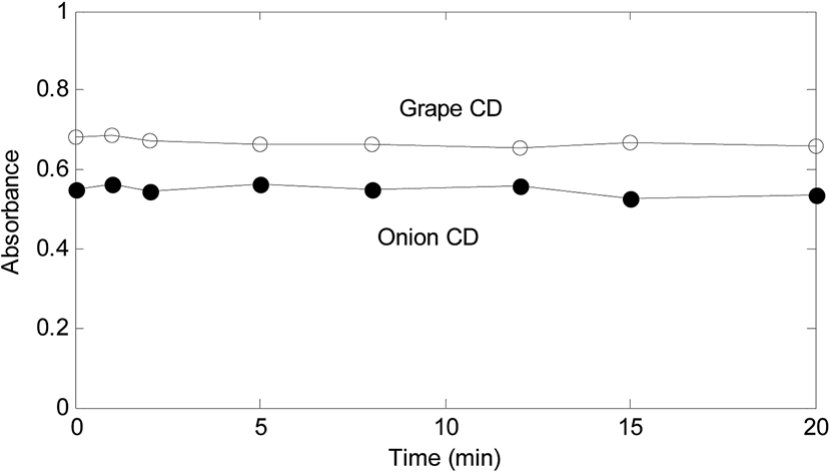
Absorbance–time plots for contact of Fe^3+^ with the as-synthesized CDs.

A critical step of the proposed method is reduction of Fe^3+^ by CDs. In order to explore about the significance of time on the reduction, in a series of solutions containing Fe^3+^ (3.00 × 10^−5^ mol L^−1^), a fixed amount of CDs (1700.0 mg L^−1^) were added and mixed well. After different contact times, 1,10-phenanthroline (9.00 × 10^−3^ mol L^−1^) was added to all solutions. Then, spectrum of each solution was recorded. Fig. 3 shows the resulted absorbance-time graphs at 510 nm.

As absorbance–time plots in Fig. 3 show, time does not have a significant effect of the response for both synthesized CDs. However, for assurance of the completeness of the reduction and saving time, 2 min was selected as the time for contact of Fe^3+^ with CDs.

### 3.4. Analysis of variance (ANOVA) of the designed experiments

RSM, a well matured design of experiment method, is frequently used for the optimization of chemical reactions and/or industrial processes.

The relation between the response and the factors can be shown by the following polynomial equation:*Y* = *b*_0_ + *b*_1_*x*_1_ + *b*_2_*x*_2_ + *b*_11_*x*_1_*x*_1_ + *b*_22_*x*_2_*x*_2_ + *b*_12_*x*_1_*x*_2_Here, *b*_0_ is the constant in the model and equals to the mean response of the experiments, *b*_1_ and *b*_2_ are the coefficients of linear terms, *b*_11_ and *b*_22_ are the coefficients of the square terms and *b*_12_ is the coefficient of the interaction term. The coefficients of the above model can be calculated and significance of different terms can be statistically evaluated by analysis of variance (ANOVA) of the experiments in Table S1.† The results of ANOVA have been given in Table 1.

**Table 1:** Results of ANOVA for the designed experiments in Table S1

Term	Coefficient	*t*	*p*
**Grape CD**			
Constant	0.635	13.3	0.000
*x*_1_	0.202	5.1	0.001
*x*_2_	0.160	4.2	0.004
*x*_1_ *x*_1_	−0.189	−4.3	0.004
*x*_2_ *x*_2_	−0.058	−1.4	0.200
*x*_1_ *x*_2_	0.092	1.7	0.128

**Onion CD**			
Constant	0.617	9.3	0.000
*x*_1_	0.153	2.8	0.028
*x*_2_	0.174	3.3	0.014
*x*_1_ *x*_1_	−0.173	−2.8	0.028
*x*_2_ *x*_2_	−0.066	−1.1	0.290
*x*_1_ *x*_2_	0.038	0.5	0.625

Column “Coefficient” in Table 1 includes the coefficient of each term in the above polynomial equation. Sign of these coefficients is important. For example, sign of the coefficients for *x*_1_ and *x*_2_ terms in the case of onion and grape CDs are positive. This shows that by increasing the concentration of CDs (*x*_1_) and 1,10-phenanthroline (*x*_2_), the response is increased. The columns “*t*” and “*p*” show the statistical significance of different terms. The larger the *t* statistics, the higher the significance of the corresponding term. The *p* value indicates the probability of the effect of chance in the importance of each term. Therefore, lower *p* values indicate that the corresponding term is significant. From Table 1, it can be concluded that concentration of CDs (*x*_1_) and 1,10-phenanthroline (*x*_2_) are significant factors in the reaction of Fe^3+^ with 1,10-phenanthroline in the presence of both CDs since corresponding *p* values are very small (<0.05, testing at 95% confidence level). Moreover, coefficients of these terms in the model are positive. This means that higher concentrations of 1,10-phenanthroline and CDs result in the higher responses. Square terms relating the concentration of CDs are also significant. Therefore, it would be observed that the effect of this factor be dependent on its level.

**Fig. 4:**
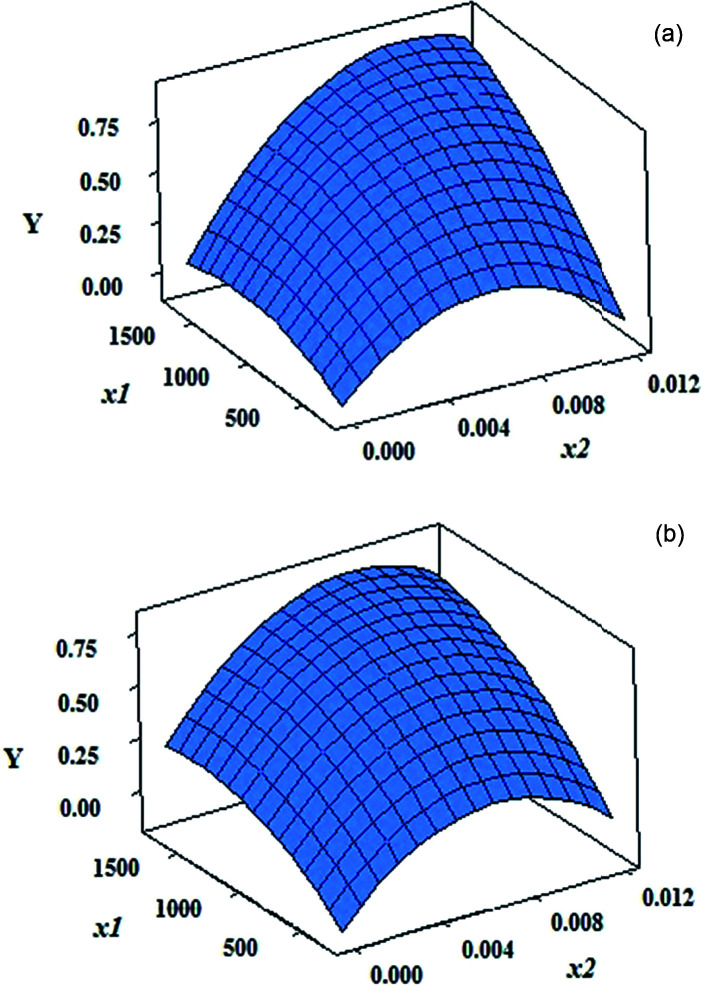
Response surfaces obtained based on the models with the coefficients reported in Table 1 for concentration of CDs and 1,10-phenanthroline by (a) grape CDs and (b) onion CDs.

Fig. 4 shows the variation of the response with simultaneous change in the level of two factors. As can be seen, response surfaces are very similar in the presence of grape and onion CDs. Curvature in the response surface upon change in the level of *x*_2_ in both cases is evident. It can be seen that in higher concentrations of CDs and relatively high concentrations of 1,10-phenanthroline, the response is higher.

Response surface optimization showed that, in the presence of the both CDs, the maximum response can be achieved by 1700.0 mg L^−1^ of CD and 9.00 × 10^−3^ mol L^−1^ of 1,10-phenanthroline. These amounts can readily been deduced from the response surfaces in Fig. 4.

### 3.5. Determination of total iron and Fe^3+^ using CDs as reducing agent

As it was shown in Section 3.2, the as synthesized CDs can act as reducing agents. Therefore, the synthesized CDs were employed as reducing agent in determination of Fe^3+^ by 1,10-phenanthroline.

Here, the analytical calibration curves were obtained by using CDs as reducing agent for Fe^3+^ and 1,10-phenanthroline as complexing agent in optimal conditions. In the presence of different concentrations of Fe^3+^, absorbances of the mixture of CDs and 1,10-phenanthroline were recorded at 510 nm. The analytical characteristics of the calibration curves have been included in Table S2.†

The statistical parameters in Table S2† show that determination of Fe^3+^ with two synthesized CDs can be performed with similar sensitivity (LODs: 1.0 × 10^−7^ and 1.2 × 10^−7^ and slopes: 7484.4 and 6732.7 by grape and onion CDs, respectively). *F*-Statistics of the two calibration curves are very high (5607.7 and 3471.9 for grape and onion CDs, respectively) which indicate that variation in the response is significantly due to the change in concentration of Fe^3+^. In Table S2,† the analytical characteristics of the calibration of Fe^2+^ in the presence of 1,10-phenanthroline have also been reported. As is expected, characteristics indicating the sensitivity like slope are higher relative to those obtained by calibration of Fe^3+^-CD in the presence of 1,10-phenanthroline. The slopes for calibration of Fe^3+^ in the presence of 1,10-phenanthroline for grape and onion CDs are 7484.4 and 6732.7, respectively and for Fe^2+^ in the presence of 1,10-phenanthroline is 8293.7. In fact, the ratio between the slopes by Fe^3+^ and by Fe^2+^ can be considered as an estimate of the Fe^3+^ to Fe^2+^ conversion for each CD.

**Fig. 5:**
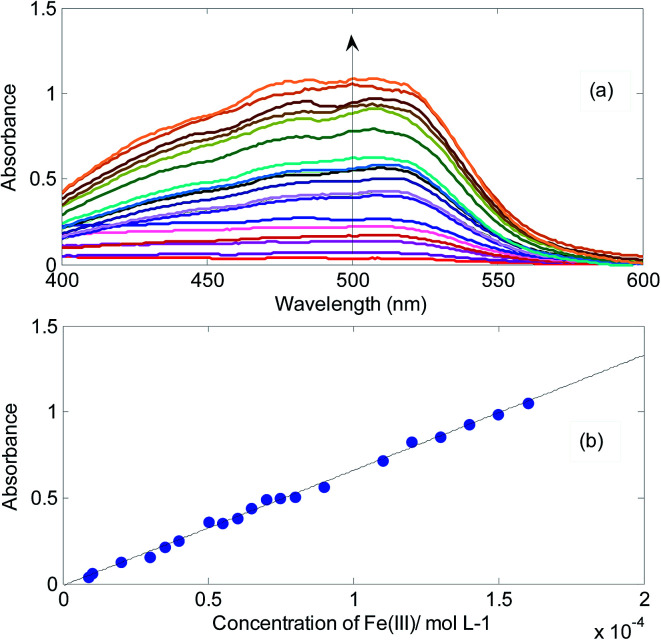
(a) Spectra of the solutions containing onion CDs (1700 mg L^−1^), 1,10-phenanthroline (9.00 × 10^−3^ mol L^−1^) and different concentrations of Fe^3+^ and (b) calibration curve constructed by absorbances at 510 nm. Arrow shows the direction of absorbance changes upon addition of Fe^3+^.

In Fig. 5, absorbance changes of the mixture of onion CDs and 1,10-phenanthroline in the presence of different concentrations of Fe^3+^ and corresponding calibration curve have been shown. The emergence of the absorption band is due to the reduction of Fe^3+^ to Fe^2+^ and subsequent formation of complex between Fe^2+^ and 1,10-phenanthroline.

Fe^3+^ is the predominate species of iron in the natural water samples. Hence, determination of total iron based on an analysis for Fe^2+^ requires firstly reduction of Fe^3+^ to Fe^2+^. Here, CDs were introduced as reducing agent in this step.

Results of the analysis of different water samples by the proposed method have been included in Table 2. Concentration of Fe^2+^ in real samples was calculated based on an independent calibration curve constructed by plotting absorbances at 510 nm of acidic hydrochloric solution (0.5 mol L^−1^) of 1,10-phenanthroline (0.01 mol L^−1^) after addition of different amounts of Fe^2+^ in the absence of CDs. Analyzing the same samples but now in the presence of CDs would give the total iron content (Fe^2+^ plus Fe^3+^) of the sample. For obtaining concentration of Fe^3+^ in the analyzed samples, it is only needed to subtract the result in the absence of CDs from the result in the presence of CDs.

Results of the analysis of different water samples by the proposed method. For each analysis, six replicates have been performed

**Table:** 

Sample	Grape CD				Onion CD				
	Added (Fe^3+^)	Found	RE^a^ (%)	RSD^b^ (%)	Added (Fe^3+^)	Found	RE%	RSD%	AA (ppb)
**Tap water**									
	0.00	N.D.^c^	—	—	0.00	N.D.	—	—	4.5
	5.00 × 10^−5^	5.18 × 10^−5^	3.7	4.7	6.00 × 10^−5^	5.85 × 10^−5^	−2.3	5.2	

**Niloufar mirage water**									
	0.00	N.D.	—	—	0.00	N.D.	—	—	6.6
	5.00 × 10^−5^	4.56 × 10^−5^	−8.7	6.5	6.00 × 10^−5^	5.80 × 10^−5^	−3.2	8.2	

**Qar-e-Sou River water**									
	0.00	N.D.	—	—	0.00	N.D.	—	—	7.0
	5.00 × 10^−5^	4.84 × 10^−5^	−3.2	2.0	6.00 × 10^−5^	5.50 × 10^−5^	−7.7	4.8	

a Relative error of prediction.

b Relative standard deviation.

c Not detected.

**Table:** 

Spiked deionized water	Added (Fe^3+^)	Found (Fe^3+^)	Added (total iron)	Found (total iron)
**Grape CD**				
	3.00 × 10^−5^	3.10 × 10^−5^	6.00 × 10^−5^	6.35 × 10^−5^
RSD%		6.5		2.9

**Onion CD**				
	3.00 × 10^−5^	3.12 × 10^−5^	6.00 × 10^−5^	6.38 × 10^−5^
RSD%		6.4		2.9

In the analyzed real water samples, it was not detected any iron species by the proposed method. However, analysis of the spiked samples resulted in satisfactory statistics for accuracy and precision (RE% and RSD% values are below 10%). For validating the proposed method, the same samples were also analyzed by standard method of atomic absorption spectroscopy. By the standard method, total iron contents of the samples were obtained in the ppb level which is below the determination level of the proposed method.

Water samples containing known amounts of Fe^3+^ and Fe^2+^ were also selected to analysis by the proposed method. Results of analysis of these samples have also been reported in Table 2. As can be deduced from the results in Table 2, total iron and Fe^3+^ concentration in these samples have been recovered with low RE% and RSD% values. Relatively low values of RSD% indicate that the proposed method is reproducible.

### 3.6. Reducing strength of CDs estimated by PB assay

In order to estimate the concentration of total reducing compounds in foods which mainly is due to the presence of phenolic compounds, the Prussian blue method has been introduced.^35,36^ The intensity of the spectrophotometric signal of the assay is linearly correlated to the level of reducing substances.

The estimated reducing strengths of the grape and onion CDs are 25.50 and 21.94 mg of gallic acid per g of the analyte, respectively. This means that for example, 1.0 g of the grape CD have a reducing strength equivalent to 25.50 mg of gallic acid. Moreover, reducing strength of grape CD is higher than reducing strength of onion CD. Higher reducing strength of grape CD was also deduced by comparison of the slope of the calibration curves in Section 3.5.

## 4. Conclusions

It was found that CDs prepared by grape and onion have reducing ability. In comparison with other reducing agents, CDs are superior because they are environment friendly and biocompatible. Moreover, in the present work, for preparation of reducing CDs, a green and simple method without using any toxic substances was used. Using CDs is recommended in analyses where a reduction is required. A sequential approach for determination of Fe^2+^ and Fe^3+^ was proposed based on the reducing CDs. These green reducing agents can be incorporated in the previous procedures for sequential determination of Fe^2+^ and Fe^3+^ which are based on the reduction of Fe^3+^. Reducing strength of the synthesized CDs can be estimated by PB assay.

## Conflicts of interest

There are no conflicts to declare.

## Supplementary Material

RA-008-C7RA12139C-s001
