# Supplementary material for: Application of response surface methodology and green carbon dots as reducing agents in speciation of iron
Source: RSC Adv. 2018 Jan 9;8(4):2173–80. doi: 10.1039/c7ra12139c (PMC9077211; doi:10.1039/c7ra12139c)
Supplement: RA-008-C7RA12139C-s001 [file RA-008-C7RA12139C-s001.pdf]

Electronic Supplementary Material (ESI) for RSC Advances.
This journal is © The Royal Society of Chemistry 2018

Application of response surface methodology and green carbon dots as reducing agent in speciation of iron

Masoud Shariati-Rad*, Tahereh Mohseninasab, Fatemeh Parno

Department of Analytical Chemistry, Faculty of Chemistry, Razi University, Kermanshah, Iran

*Corresponding author: Fax: (+ 98) 833 4274559, E– mail: mshariati_rad@yahoo.com

Table S1

Experiments designed based on RSM for exploring the influence of concentration of CDs and 1,10-phenanthroline on the complex formation between Fe^{3+} (5.00×10^{-5} mol L^{-1}) and 1,10-phenanthroline.

Experiment NO.	Concentration of CD (mg L^{-1})	Concentration of 1,10-phenanthroline (mol L^{-1})	Response	
			Grape CD	Onion CD
1	300.0	0.0055	0.287	0.188
2	1700.0	0.0055	0.761	0.649
3	500.0	0.0010	0.206	0.256
4	500.0	0.0100	0.263	0.275
5	1000.0	0.0055	0.623	0.609
6	1000.0	0.0055	0.567	0.589
7	1000.0	0.0055	0.668	0.647
8	1000.0	0.0000	-0.035	-0.097
9	1000.0	0.0055	0.753	0.678
10	1000.0	0.0119	0.652	0.552
11	1000.0	0.0055	0.602	0.632
12	1500.0	0.0010	0.325	0.545
13	1500.0	0.0100	0.750	0.717

Table S2

Analytical characteristics of the calibration curves of the Fe^{3+}-1,10-phenanthroline system in the presence of grape and onion CDs.

Parameter			
	Fe^{3+}-Grape CD	Fe^{3+}-Onion CD	Fe^{2+}
Linear range (mol L^{-1})	4.6×10^{-6}-1.6×10^{-4}	9.0×10^{-6}-1.6×10^{-4}	6.0×10^{-6}-1.8×10^{-4}
Limit of detection (LOD) (mol L^{-1})	1.0×10^{-7}	1.2×10^{-7}	9.6×10^{-8}
Slope	7484.4	6732.7	8293.7
Intercept	0.02	-0.01	-0.066
Correlation coefficient (r)	0.998	0.994	0.998
F-statistics	5607.7	3471.9	10910.3

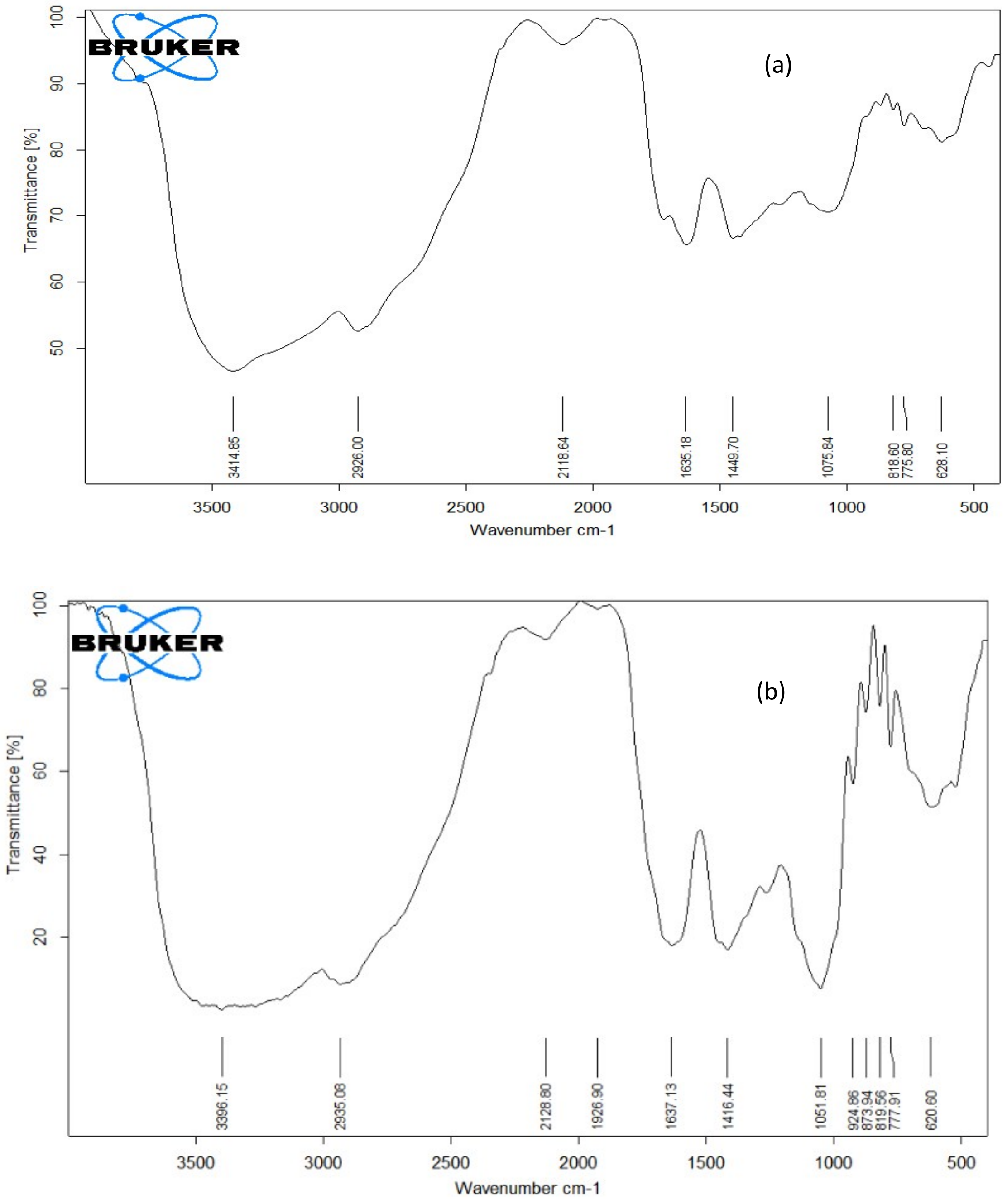

Fig. 1S. FTIR spectrum of as-synthesized (a) grape CD and (b) onion CD.
